# Supplementary material for: Heparin-based hydrogel scaffolding alters the transcriptomic profile and increases the chemoresistance of MDA-MB-231 triple-negative breast cancer cells
Source: Biomater Sci. 2020 Feb 13;8(10):2786–96. doi: 10.1039/c9bm01481k (PMC7497406; doi:10.1039/c9bm01481k)
Supplement: Supplementary file 2 [file BM-008-C9BM01481K-s002.zip › Supplementary File 4/HP-BhydrogelvControl/Pathways/my_analysis.Gsea.1545201079907/HALLMARK_HEME_METABOLISM.html]

Details for gene set HALLMARK\_HEME\_METABOLISM[GSEA]

|  || Dataset | expr.class.cls#HYD\_versus\_CONTROL.class.cls#HYD\_versus\_CONTROL\_repos |
| Phenotype | class.cls#HYD\_versus\_CONTROL\_repos |
| Upregulated in class | CONTROL |
| GeneSet | HALLMARK\_HEME\_METABOLISM |
| Enrichment Score (ES) | -0.39160344 |
| Normalized Enrichment Score (NES) | -1.8597363 |
| Nominal p-value | 0.0 |
| FDR q-value | 0.0016153421 |
| FWER p-Value | 0.005 |
Table: GSEA Results Summary

  

Fig 1: Enrichment plot: HALLMARK\_HEME\_METABOLISM      
 Profile of the Running ES Score & Positions of GeneSet Members on the Rank Ordered List

  

| PROBE | DESCRIPTION (from dataset) | GENE SYMBOL | GENE\_TITLE | RANK IN GENE LIST | RANK METRIC SCORE | RUNNING ES | CORE ENRICHMENT || 1 | PQLC1 | na |  |  | 171 | 3.009 | 0.0145 | No |
| 2 | RNF123 | na |  |  | 369 | 2.538 | 0.0240 | No |
| 3 | CTNS | na |  |  | 577 | 2.241 | 0.0306 | No |
| 4 | OSBP2 | na |  |  | 715 | 2.101 | 0.0398 | No |
| 5 | PIGQ | na |  |  | 756 | 2.069 | 0.0539 | No |
| 6 | RBM5 | na |  |  | 1050 | 1.838 | 0.0529 | No |
| 7 | RANBP10 | na |  |  | 1142 | 1.772 | 0.0619 | No |
| 8 | CROCCP2 | na |  |  | 1601 | 1.529 | 0.0498 | No |
| 9 | ANK1 | na |  |  | 1670 | 1.502 | 0.0580 | No |
| 10 | SLC22A4 | na |  |  | 2621 | 1.137 | 0.0169 | No |
| 11 | MAP2K3 | na |  |  | 2628 | 1.135 | 0.0255 | No |
| 12 | MARCH8 | na |  |  | 2719 | 1.111 | 0.0294 | No |
| 13 | PPP2R5B | na |  |  | 3034 | 1.028 | 0.0210 | No |
| 14 | SLC6A8 | na |  |  | 3116 | 1.006 | 0.0246 | No |
| 15 | MFHAS1 | na |  |  | 3224 | 0.982 | 0.0266 | No |
| 16 | ALDH1L1 | na |  |  | 3254 | 0.976 | 0.0327 | No |
| 17 | AGPAT4 | na |  |  | 3474 | 0.923 | 0.0284 | No |
| 18 | FOXO3 | na |  |  | 3688 | 0.873 | 0.0240 | No |
| 19 | KHNYN | na |  |  | 3888 | 0.835 | 0.0201 | No |
| 20 | ATP6V0A1 | na |  |  | 3947 | 0.823 | 0.0235 | No |
| 21 | PSMD9 | na |  |  | 4087 | 0.792 | 0.0224 | No |
| 22 | SPTB | na |  |  | 4126 | 0.784 | 0.0265 | No |
| 23 | E2F2 | na |  |  | 4167 | 0.776 | 0.0305 | No |
| 24 | ABCG2 | na |  |  | 4817 | 0.655 | 0.0015 | No |
| 25 | SYNJ1 | na |  |  | 4862 | 0.647 | 0.0042 | No |
| 26 | H1F0 | na |  |  | 5247 | 0.593 | -0.0113 | No |
| 27 | ERMAP | na |  |  | 5278 | 0.588 | -0.0083 | No |
| 28 | TMCC2 | na |  |  | 5393 | 0.571 | -0.0098 | No |
| 29 | PPOX | na |  |  | 5405 | 0.569 | -0.0060 | No |
| 30 | MARK3 | na |  |  | 5609 | 0.537 | -0.0124 | No |
| 31 | ABCB6 | na |  |  | 5752 | 0.519 | -0.0158 | No |
| 32 | TSPO2 | na |  |  | 5771 | 0.517 | -0.0128 | No |
| 33 | RHD | na |  |  | 6109 | 0.470 | -0.0268 | No |
| 34 | RAP1GAP | na |  |  | 6262 | 0.449 | -0.0313 | No |
| 35 | BMP2K | na |  |  | 6345 | 0.437 | -0.0322 | No |
| 36 | SMOX | na |  |  | 6502 | 0.415 | -0.0371 | No |
| 37 | MARCH2 | na |  |  | 6865 | 0.375 | -0.0532 | No |
| 38 | NARF | na |  |  | 7023 | 0.358 | -0.0587 | No |
| 39 | SIDT2 | na |  |  | 7049 | 0.354 | -0.0572 | No |
| 40 | TNS1 | na |  |  | 7080 | 0.351 | -0.0560 | No |
| 41 | CPOX | na |  |  | 7197 | 0.339 | -0.0595 | No |
| 42 | C3 | na |  |  | 7510 | 0.306 | -0.0735 | No |
| 43 | PGLS | na |  |  | 7549 | 0.300 | -0.0732 | No |
| 44 | FBXO9 | na |  |  | 7596 | 0.294 | -0.0733 | No |
| 45 | BTG2 | na |  |  | 7702 | 0.284 | -0.0766 | No |
| 46 | SLC25A37 | na |  |  | 7868 | 0.265 | -0.0832 | No |
| 47 | TRAK2 | na |  |  | 7991 | 0.249 | -0.0876 | No |
| 48 | FN3K | na |  |  | 8105 | 0.246 | -0.0916 | No |
| 49 | HMBS | na |  |  | 8235 | 0.228 | -0.0966 | No |
| 50 | EZH1 | na |  |  | 8273 | 0.222 | -0.0969 | No |
| 51 | SEC14L1 | na |  |  | 8319 | 0.218 | -0.0975 | No |
| 52 | MPP1 | na |  |  | 8785 | 0.168 | -0.1206 | No |
| 53 | HDGF | na |  |  | 8798 | 0.167 | -0.1200 | No |
| 54 | UBAC1 | na |  |  | 9055 | 0.147 | -0.1323 | No |
| 55 | GCLC | na |  |  | 9105 | 0.140 | -0.1338 | No |
| 56 | AQP3 | na |  |  | 9407 | 0.104 | -0.1488 | No |
| 57 | RCL1 | na |  |  | 9619 | 0.086 | -0.1592 | No |
| 58 | NFE2L1 | na |  |  | 9663 | 0.078 | -0.1608 | No |
| 59 | NFE2 | na |  |  | 9732 | 0.072 | -0.1638 | No |
| 60 | TSPAN5 | na |  |  | 9789 | 0.066 | -0.1663 | No |
| 61 | BCAM | na |  |  | 9832 | 0.060 | -0.1680 | No |
| 62 | TFDP2 | na |  |  | 9840 | 0.060 | -0.1679 | No |
| 63 | HBQ1 | na |  |  | 9867 | 0.056 | -0.1688 | No |
| 64 | HAGH | na |  |  | 9900 | 0.055 | -0.1701 | No |
| 65 | EPOR | na |  |  | 9957 | 0.045 | -0.1727 | No |
| 66 | IGSF3 | na |  |  | 10027 | 0.037 | -0.1760 | No |
| 67 | RAD23A | na |  |  | 10165 | 0.019 | -0.1831 | No |
| 68 | TNRC6B | na |  |  | 10171 | 0.018 | -0.1832 | No |
| 69 | SLC6A9 | na |  |  | 10362 | 0.011 | -0.1931 | No |
| 70 | KAT2B | na |  |  | 10387 | 0.008 | -0.1943 | No |
| 71 | SLC30A10 | na |  |  | 10400 | 0.007 | -0.1948 | No |
| 72 | ARHGEF12 | na |  |  | 10414 | 0.006 | -0.1955 | No |
| 73 | SLC11A2 | na |  |  | 10509 | -0.006 | -0.2004 | No |
| 74 | TFRC | na |  |  | 10516 | -0.007 | -0.2006 | No |
| 75 | ADD1 | na |  |  | 10759 | -0.033 | -0.2131 | No |
| 76 | P4HA2 | na |  |  | 10841 | -0.045 | -0.2170 | No |
| 77 | LRP10 | na |  |  | 11043 | -0.071 | -0.2270 | No |
| 78 | GCLM | na |  |  | 11055 | -0.073 | -0.2270 | No |
| 79 | NR3C1 | na |  |  | 11080 | -0.075 | -0.2277 | No |
| 80 | RBM38 | na |  |  | 11097 | -0.079 | -0.2279 | No |
| 81 | DCAF11 | na |  |  | 11137 | -0.084 | -0.2293 | No |
| 82 | NEK7 | na |  |  | 11301 | -0.089 | -0.2372 | No |
| 83 | EPB41 | na |  |  | 11440 | -0.106 | -0.2436 | No |
| 84 | CCND3 | na |  |  | 11490 | -0.109 | -0.2453 | No |
| 85 | OPTN | na |  |  | 11715 | -0.140 | -0.2560 | No |
| 86 | SELENBP1 | na |  |  | 11758 | -0.146 | -0.2571 | No |
| 87 | SLC10A3 | na |  |  | 11809 | -0.151 | -0.2585 | No |
| 88 | DAAM1 | na |  |  | 11853 | -0.158 | -0.2595 | No |
| 89 | BTRC | na |  |  | 11891 | -0.162 | -0.2602 | No |
| 90 | PC | na |  |  | 12058 | -0.180 | -0.2675 | No |
| 91 | FOXJ2 | na |  |  | 12083 | -0.184 | -0.2673 | No |
| 92 | CTSE | na |  |  | 12424 | -0.222 | -0.2835 | No |
| 93 | ATG4A | na |  |  | 12913 | -0.273 | -0.3070 | No |
| 94 | TMEM9B | na |  |  | 12927 | -0.274 | -0.3055 | No |
| 95 | SLC7A11 | na |  |  | 12949 | -0.278 | -0.3045 | No |
| 96 | GLRX5 | na |  |  | 12991 | -0.283 | -0.3044 | No |
| 97 | SLC30A1 | na |  |  | 13404 | -0.338 | -0.3234 | No |
| 98 | HIST1H4C | na |  |  | 13659 | -0.379 | -0.3338 | No |
| 99 | UCP2 | na |  |  | 13665 | -0.379 | -0.3311 | No |
| 100 | ALAD | na |  |  | 13731 | -0.393 | -0.3314 | No |
| 101 | RNF19A | na |  |  | 14034 | -0.422 | -0.3440 | No |
| 102 | NNT | na |  |  | 14077 | -0.428 | -0.3429 | No |
| 103 | BSG | na |  |  | 14079 | -0.428 | -0.3396 | No |
| 104 | MOCOS | na |  |  | 14108 | -0.429 | -0.3377 | No |
| 105 | SLC25A38 | na |  |  | 14338 | -0.469 | -0.3461 | No |
| 106 | MKRN1 | na |  |  | 14358 | -0.471 | -0.3434 | No |
| 107 | PDZK1IP1 | na |  |  | 14704 | -0.524 | -0.3574 | No |
| 108 | GAPVD1 | na |  |  | 14707 | -0.524 | -0.3534 | No |
| 109 | SLC2A1 | na |  |  | 15114 | -0.583 | -0.3702 | No |
| 110 | BACH1 | na |  |  | 15369 | -0.629 | -0.3786 | No |
| 111 | UROS | na |  |  | 15403 | -0.635 | -0.3754 | No |
| 112 | HTATIP2 | na |  |  | 15512 | -0.653 | -0.3760 | No |
| 113 | BLVRB | na |  |  | 15546 | -0.661 | -0.3726 | No |
| 114 | FECH | na |  |  | 15638 | -0.675 | -0.3721 | No |
| 115 | CDR2 | na |  |  | 15917 | -0.727 | -0.3810 | No |
| 116 | CTSB | na |  |  | 16096 | -0.769 | -0.3844 | No |
| 117 | FBXO7 | na |  |  | 16235 | -0.796 | -0.3854 | Yes |
| 118 | TCEA1 | na |  |  | 16294 | -0.809 | -0.3821 | Yes |
| 119 | HEBP1 | na |  |  | 16319 | -0.814 | -0.3770 | Yes |
| 120 | DCUN1D1 | na |  |  | 16325 | -0.816 | -0.3709 | Yes |
| 121 | ADIPOR1 | na |  |  | 16439 | -0.840 | -0.3703 | Yes |
| 122 | DCAF10 | na |  |  | 16624 | -0.880 | -0.3731 | Yes |
| 123 | LPIN2 | na |  |  | 16674 | -0.892 | -0.3687 | Yes |
| 124 | GMPS | na |  |  | 16723 | -0.905 | -0.3641 | Yes |
| 125 | HTRA2 | na |  |  | 16878 | -0.948 | -0.3648 | Yes |
| 126 | FBXO34 | na |  |  | 17243 | -1.062 | -0.3757 | Yes |
| 127 | CAST | na |  |  | 17324 | -1.092 | -0.3713 | Yes |
| 128 | TOP1 | na |  |  | 17369 | -1.108 | -0.3650 | Yes |
| 129 | ARL2BP | na |  |  | 17398 | -1.118 | -0.3577 | Yes |
| 130 | BLVRA | na |  |  | 17422 | -1.122 | -0.3502 | Yes |
| 131 | ENDOD1 | na |  |  | 17504 | -1.156 | -0.3454 | Yes |
| 132 | NUDT4 | na |  |  | 17547 | -1.176 | -0.3384 | Yes |
| 133 | MBOAT2 | na |  |  | 17576 | -1.188 | -0.3306 | Yes |
| 134 | CIR1 | na |  |  | 17605 | -1.200 | -0.3227 | Yes |
| 135 | EIF2AK1 | na |  |  | 17616 | -1.205 | -0.3138 | Yes |
| 136 | PRDX2 | na |  |  | 17621 | -1.207 | -0.3046 | Yes |
| 137 | CLCN3 | na |  |  | 17722 | -1.250 | -0.3001 | Yes |
| 138 | XPO7 | na |  |  | 17754 | -1.263 | -0.2919 | Yes |
| 139 | GDE1 | na |  |  | 17761 | -1.266 | -0.2823 | Yes |
| 140 | ELL2 | na |  |  | 17763 | -1.268 | -0.2724 | Yes |
| 141 | MINPP1 | na |  |  | 17781 | -1.278 | -0.2633 | Yes |
| 142 | USP15 | na |  |  | 17862 | -1.315 | -0.2573 | Yes |
| 143 | KLF3 | na |  |  | 17909 | -1.336 | -0.2492 | Yes |
| 144 | XK | na |  |  | 17911 | -1.337 | -0.2389 | Yes |
| 145 | LMO2 | na |  |  | 18032 | -1.403 | -0.2342 | Yes |
| 146 | RIOK3 | na |  |  | 18045 | -1.411 | -0.2238 | Yes |
| 147 | PICALM | na |  |  | 18183 | -1.488 | -0.2194 | Yes |
| 148 | MGST3 | na |  |  | 18186 | -1.490 | -0.2079 | Yes |
| 149 | ASNS | na |  |  | 18216 | -1.509 | -0.1976 | Yes |
| 150 | YPEL5 | na |  |  | 18282 | -1.552 | -0.1889 | Yes |
| 151 | UROD | na |  |  | 18338 | -1.593 | -0.1793 | Yes |
| 152 | NCOA4 | na |  |  | 18359 | -1.611 | -0.1678 | Yes |
| 153 | CDC27 | na |  |  | 18397 | -1.639 | -0.1569 | Yes |
| 154 | ALDH6A1 | na |  |  | 18402 | -1.641 | -0.1443 | Yes |
| 155 | VEZF1 | na |  |  | 18482 | -1.693 | -0.1353 | Yes |
| 156 | CLIC2 | na |  |  | 18495 | -1.703 | -0.1226 | Yes |
| 157 | BPGM | na |  |  | 18542 | -1.737 | -0.1114 | Yes |
| 158 | MXI1 | na |  |  | 18597 | -1.777 | -0.1004 | Yes |
| 159 | CCDC28A | na |  |  | 18603 | -1.779 | -0.0868 | Yes |
| 160 | MOSPD1 | na |  |  | 18656 | -1.853 | -0.0750 | Yes |
| 161 | BNIP3L | na |  |  | 18688 | -1.884 | -0.0619 | Yes |
| 162 | SDCBP | na |  |  | 18755 | -1.959 | -0.0501 | Yes |
| 163 | ISCA1 | na |  |  | 18884 | -2.139 | -0.0401 | Yes |
| 164 | FAM46C | na |  |  | 18953 | -2.269 | -0.0260 | Yes |
| 165 | LAMP2 | na |  |  | 19026 | -2.430 | -0.0108 | Yes |
| 166 | CAT | na |  |  | 19052 | -2.529 | 0.0077 | Yes |
Table: GSEA details [plain text format]

  

Fig 2: HALLMARK\_HEME\_METABOLISM      
 Blue-Pink O' Gram in the Space of the Analyzed GeneSet

  

Fig 3: HALLMARK\_HEME\_METABOLISM: Random ES distribution      
 Gene set null distribution of ES for **HALLMARK\_HEME\_METABOLISM**
